# Nano in Implant Dentistry

**DOI:** 10.1155/2014/314819

**Published:** 2014-03-06

**Authors:** Ryo Jimbo, Martin Andersson, Stefan Vandeweghe

**Affiliations:** ^1^Department of Prosthodontics, Faculty of Odontology, Malmö University, 205 06 Malmö, Sweden; ^2^Department of Chemical and Biological Engineering, Applied Surface Chemistry, Chalmers University of Technology, 412 96 Gothenburg, Sweden; ^3^Department of Periodontology and Oral Implantology, Dental School, Faculty of Medicine and Health Sciences, University of Ghent, 9000 Ghent, Belgium

Nanolevel modification of biomaterials has significantly improved their properties. With regard to implant dentistry, the application of “nano” to implants, abutments, and bone substitutes drastically changed the biologic response to them. Numerous in vitro, in vivo, and clinical studies proved that the submicron modification, normally impossible to detect with our eyes, has enhanced osteogenesis to the biomaterial. An interesting fact is that one of the authors in this special issue L. M. Svanborg has suggested in her thesis work that nanotopography in fact exists on most of the commercially available implant surfaces. However, these structures are most of the time the result of native titanium oxides formed due to the exposure to air; thus, these structures are regarded as nonintended nanomodification. 

An important aspect for the application of “nano” to biomaterial surfaces is that the modification is “intended” or “strategically” performed. Cell responses differ depending on the topography and chemistry, and controlling these parameters could target specific biologic phenomena. Thus, studies show that not only the successful enhancement of osseointegration but also the enhancement of soft tissue responses to abutment surfaces. 

This special issue is a comprehensive compendium of the state of the art research directed towards implants dentistry. A. Alenezi et al. compared two different commercially available implant surfaces, one with and one without nanostructures. It was suggested that the two different surfaces present distinct bone healing kinetics, and the surfaces possessing nanostructures seemed to be effective in osteoconduction at early healing periods. J. Löberg et al. have suggested that the nanostructure acts as a nucleation center for the early formation of hydroxyapatites, which supports the in vivo reports. Contradictory to these studies, the in vivo animal study performed by L. M. Svanborg et al. has shown no effects in their histological and biomechanical study. They have speculated that “nano” may not exert significant effects in sites with excellent bone quality. Furthermore, the presence of microtopography, different implant macrogeometry, and coarse evaluation techniques could be one of the causes, which could be disguising the effect of “nano.” K. Breding et al. have shown in their study that the effect of the nano-level modification could not be detected when removal torque tests were performed; however, gene expression analysis of the removed implants clearly pointed out that osteogenic gene expression had been upregulated for some genes. Based on these studies, the destructive removal torque method may not be suitable to evaluate the effect of nano-level modification and advanced techniques may be necessary for evaluation. 

Nano in implant dentistry restricts itself not only to implant surface modifications but also to other biomaterials used in this field. C. Marin et al. have shown that nanostructured synthetic alloplasts fused with collagen promoted bone ingrowth compared to other materials, which could be a promising material to regenerate bone in severe bone defects due to atrophy after tooth/teeth loss. E. Bressan et al. have shown that the antibacterial properties of silver nanoparticles are effective towards targeted pathogens, whereas no cytotoxic effects were observed on human cells. This noble nanocoating could be an effective method to apply antibacterial properties to biomaterials, such as abutment surfaces to lower the risk of biofilm formation. 

We sincerely hope that the readers will find the special issue interesting and informative, which could deepen the knowledge of the current trend in implant dentistry. 

*Ryo Jimbo* *Ryo Jimbo* *Martin Andersson* *Martin Andersson* *Stefan Vandeweghe* *Stefan Vandeweghe*

